# Toxicity of Xanthene Food Dyes by Inhibition of Human Drug-Metabolizing Enzymes in a Noncompetitive Manner

**DOI:** 10.1155/2009/953952

**Published:** 2009-08-23

**Authors:** Takaharu Mizutani

**Affiliations:** Graduate School of Pharmaceutical Sciences, Nagoya City University, Nagoya 467-8603, Japan

## Abstract

The synthetic food dyes studied were rose bengal (RB), phroxine (PL), amaranth, erythrosine B (ET), allura red, new coccine, acid red (AR), tartrazine, sunset yellow FCF, brilliant blue FCF, and indigo carmine. First, data confirmed that these dyes were not substrates for CYP2A6, UGT1A6, and UGT2B7. ET inhibited UGT1A6 (glucuronidation of p-nitrophenol) and UGT2B7 (glucuronidation of androsterone). We showed the inhibitory effect of xanthene dye on human UGT1A6 activity. Basic ET, PL, and RB in those food dyes strongly inhibited UGT1A6 activity, with IC_50_ values = 0.05, 0.04, and 0.015 mM, respectively. Meanwhile, AR of an acidic xanthene food dye showed no inhibition. Next, we studied the inhibition of CYP3A4 of a major phase I drug-metabolizing enzyme and P-glycoprotein of a major transporter by synthetic food dyes. Human CYP3A4 and P-glycoprotein were also inhibited by basic xanthene food dyes. The IC_50_ values of these dyes to inhibit CYP3A4 and P-glycoprotein were the same as the inhibition level of UGT1A6 by three halogenated xanthene food dyes (ET, PL, and RB) described above, except AR, like the results with UGT1A6 and UGT2B7. We also confirmed the noninhibition of CYP3A4 and P-gp by other synthetic food dyes. Part of this inhibition depended upon the reaction of ^1^O_2_ originating on xanthene dyes by light irradiation, because inhibition was prevented by ^1^O_2_ quenchers. We studied the influence of superoxide dismutase and catalase on this inhibition by dyes and we found prevention of inhibition by superoxide dismutase but not catalase. This result suggests that superoxide anions, originating on dyes by light irradiation, must attack drug-metabolizing enzymes. It is possible that red cosmetics containing phloxine, erythrosine, or rose bengal react with proteins on skin under lighting and may lead to rough skin.

## 1. Introduction

The study of drug metabolism started from the conjugation between glycine and benzoic acid to hippuric acid in horse urine by Wohler in 1824. He synthesized an organic compound, urea, in the first instance and also found aluminum. The study of drug metabolism has advanced according to developments in chemistry and the chemical industry since the nineteenth century; however, drug-metabolizing enzymes did not originate from chemistry development but rather were developed to excrete natural substances of low molecular weight, mainly plant materials such as catechols, terpenoids, alkaloids, flavonoids, lignins, and amines, ingested by the body with five major nutrients (carbohydrates, proteins, lipids, vitamins, and minerals) in foods from when living things were created, 3.5 billion years ago or earlier [1]. We drink coffee and juice, which contain nonnutrient materials and fibers that pass through the body as feces. Caffeine and chlorogenic acid are major constituents in coffee and are also ingested and metabolized by so-called drug-metabolizing enzymes. Many of these small materials should be excreted in urine from the kidneys and in bile from the liver after phase I and II drug-metabolizing enzymes and transporters react with them. Phase I drug-metabolizing enzyme is mainly composed of cytochrome P450 (CYP), which was identified by Omura and Sato [2]. In grapefruit juice, some unknown constituents inhibit drug metabolism by CYP3A4 of a major phase I drug-metabolizing enzyme. Of course, some drug-metabolizing enzymes play a key role in reacting with endogenous substances to be excreted, such as bilirubin and steroid hormones. Other phase I metabolizing enzymes, such as CYP11, CYP17, and CYP19, play an essential role in producing steroid hormones, such as testosterone, estradiol, aldosterone, progesterone, and corticosterones, from cholesterol through pregnenolone. The substrate specificity of these steroid synthesizing enzymes is narrow compared with that of CYPs for drug metabolism; which also produce vitamin D and retinoids. The CYP levels in human liver (about 1 kg) are 2% to 4% of the total protein; this means that the amount of CYP is approximately 5–10 g in the liver (the high level is in the case of induction by phenobarbital). Poor metabolizer frequencies of major CYPs in Asians and Caucasians have been summarized [3]. In poor metabolizers, drugs are not metabolized and high drug levels in blood are maintained, with toxic effects appearing in patients. These CYPs are present in the smooth endoplasmic reticulum and are recovered in microsomal fractions in experiments. A major phase II drug-metabolizing enzyme, UDP-glucuronosyltransferase (UGT), is also contained in microsomal fractions in the liver to the same level of CYPs. Thus, these CYPs and UGTs are major protein constituents in microsomes and a few CYPs and UGTs might be coupled and co-operate with each other in membranes.

Most xenobiotics, such as drugs, nonnutrient substances of low molecular mass in foods, and pollutants, are absorbed and then metabolized by phase I drug-metabolizing enzymes, followed by phase II enzymes, and finally excreted through transporters (phase III enzymes). Many drugs are lipophilic and persist in lipophilic membranes composed of lipid matrix. Phase I enzymes convert lipophilic drugs to potentially reactive products and make compounds less toxic [4], and then phase II drug-metabolizing enzymes conjugate with water-soluble substances, such as UDPGA for UDP-glucuronosyltransferase (UGT) [5], PAPS for sulfotransferase [6], and GSH for glutathione *S*-transferase. UGT is the most functional enzyme among phase II enzymes. Drugs, their metabolites and conjugates with glucuronic acid, sulfate, and glutathione, are excreted by transporters from the liver in bile, from the kidneys in urine and from skin in sweat. The major transporters in the liver include P-glycoprotein (P-gp), MDR-relating protein 2 (MRP2, ABCC2), Organic-anion transporting peptide 2 (OATP2), and bile salt exporting protein (BSEP). P-gp functions as a key protein of the blood-brain barrier and a major drug transporter and prevents anticancer drugs from entering in cancer cells during chemotherapy [7]. Dubin-Johnson syndrome, human jaundice, is induced by a deficiency of MRP2 [8]. These transporters operate in the plasma membrane.

 With the development of storage and manufacturing methods, processed foods constitute 60% of total foods and are increasing annually. The need for food additives is also increasing [9, 10]. These chemical food dyes are also used for coloring cosmetics and pills as well as foods. Erythrosine (ET) is used as a staining dye for dead *Schizosaccharomyces pombe* [11] and to investigate dead bacteria in human dental caries. During re-evaluation of the safety of these additives, some materials have disappeared. For example, permission to use butter yellow, an azo-dye, was withdrawn due to carcinogenicity within a year after it was granted. Twelve chemical food dyes are permitted by the Japanese Government [12]. There have been some reports showing the inhibition of enzyme activity, such as choline esterase inhibition by ET and sunset yellow, inhibition of sulfation of 17 *β*-ethinylestradiol by ET [13, 14], and inhibition of dopamine sulfation by tartrazine [15]. The inhibition of some CYPs by purpurin and alizarin has also been reported [16]. Meanwhile, amaranth has not been permitted in the USA since 1976 but is permitted in Japan. Most chemical pigments possess anionic sulfate residues that prevent the absorption of pigments in the gastrointestinal tract [10]. Some azo-dyes are reduced by enterobacteria in the intestine and are absorbed in the body [12]. Toxicity studies of these pigments in humans are difficult for many reasons, thus, toxicity studies depend on experimental results in animals [17].

 Phenyl-xanthene dyes, such as rose bengal (RB), ET, phloxine (PL), eosin (ES), uranine (UR), rhodamine (RM), and fluorescein, are known as light-enhancing reagents (catalytic light reaction) by the generation of ^1^O_2_ on the dyes [18–24]. There are two types of reaction: the first is that drug energy enhanced by light is transferred to biomolecules and free radicals originate on the molecules. The second is that energy is transferred to oxygen, which changes to ^1^O_2_. This reaction depends upon the number of halogens on xanthene dyes and the light strength. There are some papers on the inactivation of enzymes by xanthene dyes. Na,K-ATPase was inactivated by light in the presence of RB [24, 25]. Acetylcholineesterase and some microorganims, such as *Escherichia coli*, *Staphylococcus aureus*, and influenza virus, are inactivated [18, 19, 23, 26].

In this review about safety testing of human-specific drug metabolites, we showed inhibition of xanthene food dyes for drug-metabolizing enzymes, summarized from our reports [27–29]. Meanwhile, we have also investigated the induction of human UGT1A1 by bilirubin [5, 30–34], autoantibodies in autoimmune hepatitis patients [35–38], participation of human UGT1A6 in drug interaction between valproate and capbapenem antibiotics [39–41], structure-function relationships of some opioid derivatives for human UGT2B7 [42], and recent progress of the endogenous function of P-gp [43–46].

## 2. Experimental

### 2.1. Dyes

**Figure 1:**
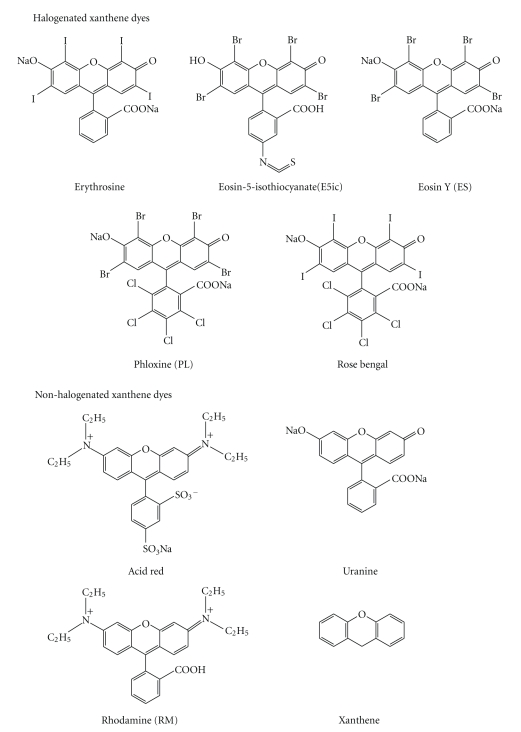
Chemical structure of xanthene food dyes.

The chemical food dyes used were phloxine (PL, Food Red no. 104), rose bengal (RB, Food Red no. 105), erythrosine (ET, Food Red no. 3), amaranth (AM, Food Red no. 2), allura red (AL, Food Red no. 40), new coccine (NC, Food Red no. 102), acid red (AR, Food Red no. 106), tartrazine (TT, Food Yellow no. 4), sunset yellow FCF (SY, Food Yellow no. 5), brilliant blue FCF (BB, Food Blue no. 1), and indigo carmine (ID, Food Blue no. 2), and parts of their structures are shown in Figure 1. These are products of San-Eigen Co. Ltd (Osaka, Japan) and have official approval for purity and safety from the Japanese Government. These dyes are well soluble in water and the solutions are used at various appropriate concentrations.

### 2.2. Drug-Metabolizing Enzymes

The enzyme source to measure CYP2A6, CYP3A4, and UGT1A6 activities was pooled human liver microsomes (HLMs), purchased from Gentest (Woburn, MA, USA). UGT2B7 and P-gp membrane are products prepared in an Sf9 cell membrane using a baculovirus expression system supplied by Gentest. These enzymes were stored at −80°C. Superoxide dismutase (SOD) and catalase are products of Sigma.

### 2.3. Assay of Drug-Metabolizing Enzymes

Coumarin 7-hydroxylation activity (CYP2A6) was measured as previously reported [47, 48] and originally [49]. The assay of CYP3A4 activity was carried out according to the method [50]. The substrate used was 7-benzoyloxy-4-(trifluoromethyl)-coumarin and the standard chemical of the product was 7-hydroxy-trifluoromethyl-coumarin, supplied by Gentest. The microassay method of UGT activity in this study was carried out according to previous reports [5, 51]. ATPase activity of P-gp is generally measured according to the protocol [52] described on the data sheet from Gentest. In order to investigate the role of reactive oxygen species on the inhibition of UGT1A6 by dyes, we studied the effect of SOD and catalase.

### 2.4. Statistical Analyses

The mean ± S.D value of each point was calculated from 3 determinations. Validity of the inhibition was examined by Student's *t*-test for differences in the presence (control) and absence of inhibitors. Significant values at the 5% level of significance were taken as effective. *Significant from the control (*P* < .05); **, (*P* < .01).

## 3. Inhibition of CYP Activity by Chemical Food Dyes

**Figure 2:**
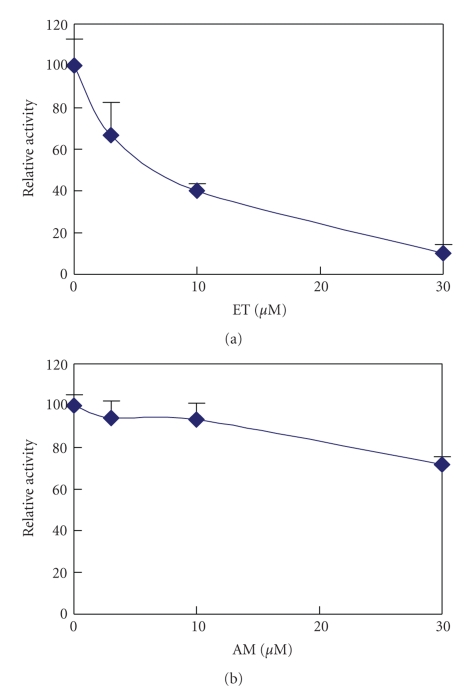
Inhibition of CYP3A4 activity by erythrosine (ET) and amaranth (AM).

CYP3A4 is a major enzyme among phase I drug-metabolizing enzymes and reacts with half of all drugs. To determine the influence of CYP3A4 activity by food dyes, the color of dyes influencing the fluorometric measurement over 30 *μ*M dye concentration was measured. This background was subtracted from the measurement of color pigments. Figure 2 shows the inhibition of CYP3A4 by ET, which was completely inhibited at 30 *μ*M, and shows noninhibition by AM. These IC_50_ values are shown in Table 1. Other xanthenes food dyes (PL, RB and AR) also showed inhibition but other food dyes did not inhibit the reaction of CYP3A4. We omitted the non-inhibition patterns of many other dyes in Table 1. As shown in Table 1, IC_50_ values for CYP3A4 reaction were similar to the values obtained by UGT1A6 reaction described later [28], except for AR, which did not inhibit the UGT1A6 reaction, but the CYP3A4. We could not explain this discrepancy.

## 4. Inhibition of UGT1A6 and UGT2B7 by Food Dyes

**Figure 3:**
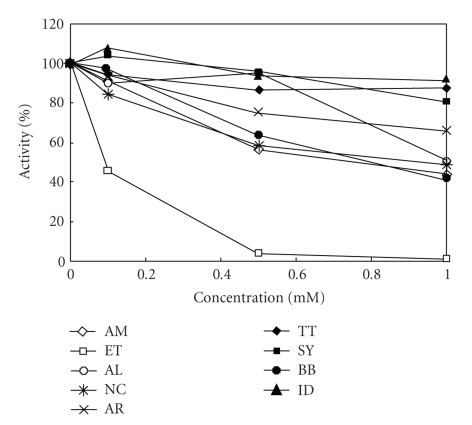
Inhibition of glucuronidation of UGT1A6 by chemical food dyes.

**Figure 4:**
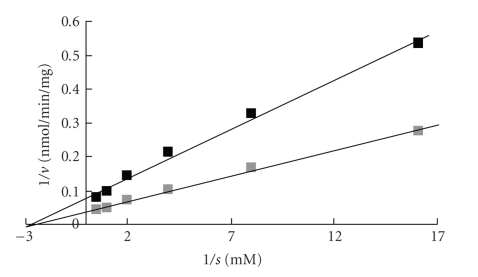
Lineweaver-Burk plots of UGT1A6 by erythrosine (ET). Closed squares and shaded squares are 0.05 mM ET and absence of ET, respectively.

The inhibition of UGT1A6 activity with p-nitrophenol [5] and of UGT2B7 with androsterone by dyes was studied. Figure 3 shows the concentration-dependent inhibition patterns of UGT1A6 by chemical food dyes. The greatest inhibition was found with ET. Dyes showing insignificant inhibition were AM, AL, NC, and BB. Pigments, such as AR, TT, SY, and IC, showed no inhibition. The results of the autoradiogram showed inhibition of UGT1A6 activity by ET in a concentration-dependent manner. The density (radioactivity) of the products became weaker relative to the ET concentration (data not shown). The result also shows that ET had no substrate activity, as did the Lineweaver-Burk plots of UGT1A6 in the presence of ET, indicating a noncompetitive manner, as shown in Figure 4. The same inhibition pattern was found in UGT2B7 with androsterone. This inhibition pattern of UGT2B7 by dyes was parallel to the pattern obtained with UGT1A6 in Figure 3. These IC_50_ values are summarized in Table 1. The IC_50_ value of inhibition by ET for UGT2B7 was similar to the value for UGT1A6. The IC_50_ values of inhibition by AM, AL, NC, BB, AR, TT, SY and ID were higher than 2 mM and showed almost no inhibition. Thus, ET, RB, PL of basic xanthenes dyes showed specific inhibition of UGT1A6 and 2B7 in a non-competitive manner.

From the structure-function relationships in glucuronidation inhibition, it is very interesting that halogenated xanthene dyes, such as ET, RB, and PL, have inhibitory activity against UGT. Thus, we studied the inhibition of UGT1A6 by other xanthene dyes, Eosin Y (ES) and Eosin-5-isothiocyanate (E5ic), of nonpermitted dyes. These dyes inhibited UGT activity and those IC_50_ values of ET, RB, PL, ES, E5ic were 0.07, 0.015, 0.04, 0.12, 0.07 mM, respectively (data not shown). At a concentration of 0.5 mM, the dyes almostly totally inhibited glucuronidation activity. Meanwhile, nonhalogenated xanthene dyes, such as AR, rhodamine, Uranine, and Xanthene, did not inhibit the activity of UGT1A6. These IC_50_ values were higher than 1 mM. From these results, we considered that halogenated-aromatic compounds should inhibit UGT1A6 activity. Next we studied inhibition by high-halogenated compounds, such as ioxaglic acid, iodixanol, meglumine iotalamate for contrast media, and sodium diatrizoate for leucocyte preparation; however, we found no inhibition using these high-halogenated compounds.

 From these results, we considered that the halogenated xanthene backbone is a key structure and iodine is the most potent element among halogens, because RB containing iodine is more potent than PL containing bromine. It is possible that the resonating double bond continuing from a carbonyl bond on the xanthene backbone is essential, as well as halogens on the xanthene backbone itself, in Figure 1. Phenyl residues on xanthene dyes may be another important residue as well as halogens on phenyl residues.

## 5. Inhibition of P-Glycoprotein Activity by Chemical Dyes

 The inhibition of P-gp by three halogenated xanthene dyes, PL, ET, and RB was confirmed. In the reaction of P-gp, inhibition by dye at 30 *μ*M was not complete and these IC_50_ values are shown in Table 1, which also shows the results of inhibition of UGT1A6 and P-gp activities. The strongest inhibitor of CYP3A4 and P-gp activities is RB as in the inhibition of UGT1A6 activity. Other dyes did not inhibit the P-gp reaction. Thus, three halogenated xanthene food dyes (ET, RB, and PL) well inhibited CYP3A4, UGT1A6, and P-gp.

CYP3A4 is the most active enzyme among phase I drug-metabolizing enzymes, and UGT1A6 is the major enzyme among phase II drug-metabolizing enzymes. P-gp is the most active enzyme among ABC transporters. Thus, three halogenated xanthene food dyes inhibited these major drug-metabolizing enzymes.

## 6. Influence of ^1^O_2_ Quenchers

The inhibition of UGT by xanthene dyes was confirmed as a non-competitive type mechanism from the pattern by Lineweaver-Burk plots, as shown in Figure 4 [27]. This indicates that inhibition relates to the velocity of the enzyme reaction and involves enzyme inactivation. In order to clarify the mechanisms, we studied the influence of ^1^O_2_ quenchers, such as NaN_3_, histidine, and *β*-carotene on glucuronidation inhibition by RB. We also investigated the influence of D_2_O on the glucuronidation reaction.

**Figure 5:**
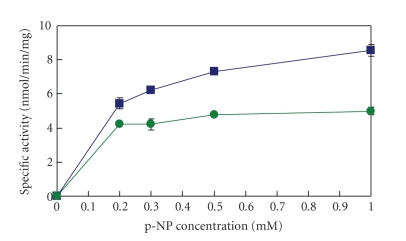
Effect of D_2_O (closed circles) and H_2_O (closed squares) on glucuronidation inhibition of p-Nitrophenol by RB.

 NaN_3_ and hisitidine significantly prevented the inhibition by RB, but *β*-carotene did not [28]. NaN_3_ and hisitidine are soluble in the reaction mixture but *β*-carotene is insoluble, so we could not obtain clear results with *β*-carotene. The prevention of RB inhibition by NaN_3_ and histidine suggests that part of the inhibition by RB depended upon ^1^O_2_ originating on RB molecules activated by light Figure 5 shows inhibition of the increase by RB in D_2_O solution and comparison of the activity in water and D_2_O, and the result shows that the activity in D_2_O is approximately half of the activity in H_2_O. This may be because part of this decrease (increase of inhibition by RB) in activity depends upon the long presence (slow disappearance) of ^1^O_2_ in D_2_O solution, as well as the slightly higher viscosity of D_2_O solution. These quenchers (NaN_3_, histidine, and *β*-carotene) themselves showed no inhibition of glucuronidation of *p*-NP by UGT1A6 in the range of concentration from 1 to 20 mM of NaN_3_ and histidine, and 0.2–0.5 mM of *β*-carotene.

 The influence of light on RB inhibition was studied. This experiment was carried out at 0.3 mM *p*-NP. We found a significant difference between the values of activity in the dark and light at low concentrations, 0.01, 0.02, and 0.05 mM of RB. This result suggests that weak inhibition by RB in the dark may depend on the low generation of ^1^O_2_ in the dark. We could not find a significant difference at a high concentration, 0.1 mM, of RB. This inhibition in the dark at 0.1 mM RB indicates that this inhibition depends on not only ^1^O_2_ but also unknown factors.

## 7. Prevention of Inhibition of UGT1A6 with SOD and Catalase

**Figure 6:**
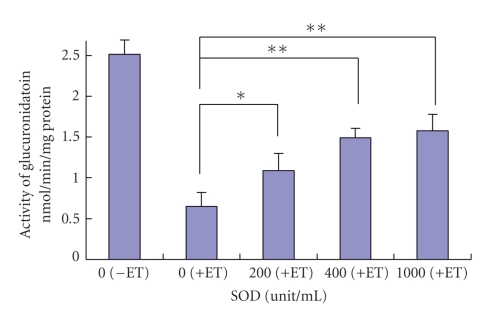
Prevention of inhibition of UGT1A6 activity by SOD of O_2_- quencher. Significance indicated by asterisk, at *P* < .05.

In order to clarify the mechanism of inhibition by dyes, we added SOD or catalase to the mixture of UGT1A6 inhibition by ET. As shown in Figure 6, the product (p-nitrophenol-glucuronide) was low in the absence of SOD, as shown on the left. The product increased according to the increase of the amount of SOD added to the inhibition mixture by ET. Prevention by SOD was significantly found in columns in the presence of SOD, showing that superoxide anions are related to inhibition by dyes. Consumption of superoxide anions by SOD recovered from the inhibition by dyes. SOD did not completely restore activity in the presence of inhibitor, possibly suggesting an alternative mechanism in addition to the free radical hypothesis. Superoxide anions may come from oxygen radicals originating on dye molecules by light irradiation.

**Figure 7:**
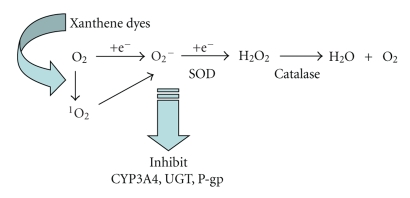
Scheme of inhibition by active oxygen species.

 We found no prevention of catalase inhibition. With a high amount of catalase, inhibition of ET was found at an identical inhibition level to that in the absence of catalase. These results indicate that hydroxyl peroxide did not relate with the inhibition of UGT1A6 activity by ET. Figure 7 shows the production pathway of active oxygen by light irradiation and a possible inhibition mechanism by dyes. Superoxide anions come from singlet oxygen and attack enzymes, such as CYP3A4, UGT1A6, and P-gp in membranes. Meanwhile, superoxide anions were hydrolyzed by SOD and inhibition by dyes was prevented by the decrease of superoxide anions by SOD.

## 8. Discussion

Chemical food additive dyes are large molecular masses having a strong anionic charge of sulfate or cationic charge on the molecule to prevent absorption in the gastrointestinal tract. It has been described that a few parts of those pigments are absorbed [12]. Approximately 2 mg total pigments/day are ingested and the concentration in the body is estimated to be 2 nM. This level is lower (1/10^5^) than the IC_50_ value of RB (0.015 mM), PL (0.04 mM) and, ET (0.05 mM) for UGT. Thus, these dyes should not influence drug metabolism and inhibition under normal conditions in the body; however, it is necessary for some patients with ulcers in the gut to avoid the ingestion of chemical food dyes. It is also possible that some cosmetics contain red xanthene dyes, activated by light irradiation, which may injure and lead to rough skin. Thus, it is recommended for a person with facial inflammation to avoid cosmetics. In the previous report [29], we showed that halogenated xanthene dyes inhibited CYP3A4, UGT1A6, and P-gp activities of major drug-metabolizing enzymes.

 It has been reported that xanthene dyes generate ^1^O_2_ in light [18–23]. The inactivation of enzymes by xanthene dyes may well proceed in aerobic conditions through type II mechanisms of ^1^O_2_ generation. It was reported that ^1^O_2_ generation on xanthene dyes is RB>ET>PL>ES≫UR [19]. By this experiment, the strength of inhibition is RB>PL>ET>E5ic>ES≫AR, RM, and UR. This order of inhibition by our study is similar to ^1^O_2_ generation. We showed that inhibition by RB was prevented by ^1^O_2_ quenchers, such as NaN_3_ and histidine [28]. The influence by *β*-carotene of another quencher was not clear and may come from the insolubility of *β*-carotene. With D_2_O, inhibition by RB was promoted, suggesting, the ^1^O_2_ played a role in the inhibition function, because of the 16-time long reservation of ^1^O_2_ in ^1^O_2_ solution [25]; however, the quencher results were not complete but partial effects in our study. The reason is that UGT1A6 is not a soluble but a membrane-bound enzyme and is buried in lipid bilayers which protect UGT1A6 from ^1^O_2_ attack. In this review, we showed that SOD prevented the inhibition of UGT1A6 activity by ET. Superoxide anions partially come from singlet oxygen, which originates on dyes by light irradiation. This result suggests that superoxide anions play a role in inhibition by dyes.

 There are few studies available for human CYP3A4 as a major phase 1 drug-metabolizing enzyme and human P-gp as a major transporter involving chemical food dyes. Many studies have been carried out on the toxicity and carcinogenicity of chemical food dyes [9, 15]. Our previous results showed that the activity levels of CYP2A6 and UGT in bovine liver microsomes were similar to human liver microsomes [5, 46, 47], but differed from rat microsomes, as rat microsomes did not involve CYP2A6 activity. Thus, it was considered that bovine microsome data were very similar to human microsome data. From the structure-function relationships in glucuronidation inhibition, it was suggested that halogenated, resonating, aromatic xanthene compounds might provide a condition for ^1^O_2_ generation to inhibit enzymes. This result suggests that superoxide anions, originating on dyes by light irradiation, must attack drug-metabolizing enzymes. It is also possible that metabolites of chemical food dyes play a role in denaturing drug-metabolizing enzymes in human microsomes, and also, red cosmetics containing phloxine, erythrosine or rose bengal react with proteins on skin under lighting by generating radicals and may lead to rough skin.

## Figures and Tables

**Table 1:** Summary of IC50 values of CYP3A4, UGT1A6, and P-glycoprotein inhibition by chemical food dyes.

Dye		IC_50_ value (*μ*M)	
	CYP3A4	UGT1A6	P-glycoprotein
Phloxine (PL)	5.6	40	24.5
Rose Bengal (RB)	21.2	15	11.7
Erythrosine B (ET)	7.9	50	15.6
Acid red (AR)	10.3	>1000	>1000
Amaranth (AM)	>1000	>1000	>1000
Allura red (AL)	>1000	>1000	>1000
New coccine (NC)	>1000	>1000	>1000
Tartrazine (TT)	>1000	>1000	>1000
Sunset yellow FCF (SY)	>1000	>1000	>1000
Brilliant blue FCF (BB)	>1000	>1000	>1000
Indigo carmine (ID)	>1000	>1000	>1000

## Abbreviations

ET: Erythrosine
PL: Phloxine
RB: Rose Bengal
CYP: Cytochrome P450
UGT: UDP-glucuronosyltransferase
P-gp: P-glycoprotein
